# Hand to Mouth in a Neandertal: Right-Handedness in Regourdou 1

**DOI:** 10.1371/journal.pone.0043949

**Published:** 2012-08-22

**Authors:** Virginie Volpato, Roberto Macchiarelli, Debbie Guatelli-Steinberg, Ivana Fiore, Luca Bondioli, David W. Frayer

**Affiliations:** 1 Department of Paleoanthropology and Messel Research, Senckenberg Research Institute Frankfurt, Frankfurt am Main, Germany; 2 Département de Préhistoire, UMR 7194, MNHN, Paris, France; 3 Département Géosciences, Université de Poitiers, Poitiers, France; 4 Department of Anthropology, The Ohio State University, Columbus, Ohio, United States of America; 5 Soprintendenza al Museo Nazionale Preistorico Etnografico “L. Pigorini”, Rome, Italy; 6 Department of Anthropology, University of Kansas, Lawrence, Kansas, United States of America; Institut de Biologia Evolutiva – Universitat Pompeu Fabra, Spain

## Abstract

We describe and analyze a Neandertal postcranial skeleton and dentition, which together show unambiguous signs of right-handedness. Asymmetries between the left and right upper arm in Regourdou 1 were identified nearly 20 years ago, then confirmed by more detailed analyses of the inner bone structure for the clavicle, humerus, radius and ulna. The total pattern of all bones in the shoulder and arm reveals that Regourdou 1 was a right-hander. Confirmatory evidence comes from the mandibular incisors, which display a distinct pattern of right oblique scratches, typical of right-handed manipulations performed at the front of the mouth. Regourdou's right handedness is consistent with the strong pattern of manual lateralization in Neandertals and further confirms a modern pattern of left brain dominance, presumably signally linguistic competence. These observations along with cultural, genetic and morphological evidence indicate language competence in Neandertals and their European precursors.

## Introduction

Various studies have identified striations on the labial (lip) face of Neandertal anterior teeth, beginning with Henri-Martin's initial observations on the upper incisors from La Quina 5 [1]. These scratches are commonly found in Neandertals from Europe [2]–[6] and in their likely ancestors from Sima de los Huesos at Atapuerca [7]–[9] and Mauer [10]. Except for two left-handed individuals, Krapina [KDP] 4 and Hortus 8, dated ∼130,000 yrs and ∼35,000 yrs, respectively, all specimens show a preponderance of right-handed striations [9]. This yields a ratio of 27 right-handed: 2 left-handed (93%: 7%), approximating the high frequencies of right-handedness found in all modern populations world-wide [9], [11]–[13]. Experimental evidence suggests these scratches were produced inadvertently as items were clenched and processed by stone tools between the canines and incisors [7], [14]. Despite earlier contentions that manipulative scratches do not appear in modern populations [15], some recent human hunter-gatherer populations show similar striations [16], [17]. However, compared to Neandertals and their European predecessors, striations are more rare in later populations. In all studies where scratches have been quantified, the marks occur only on anterior teeth, which are isolated or in jaws not directly associated with other skeletal elements. Here, we describe for the first time, to our knowledge, a Neandertal dentition, preserving multiple teeth with distinct right-handed scratches, associated with a non-pathological upper limb skeleton exhibiting unambiguous right-handedness.

**Figure 1:**
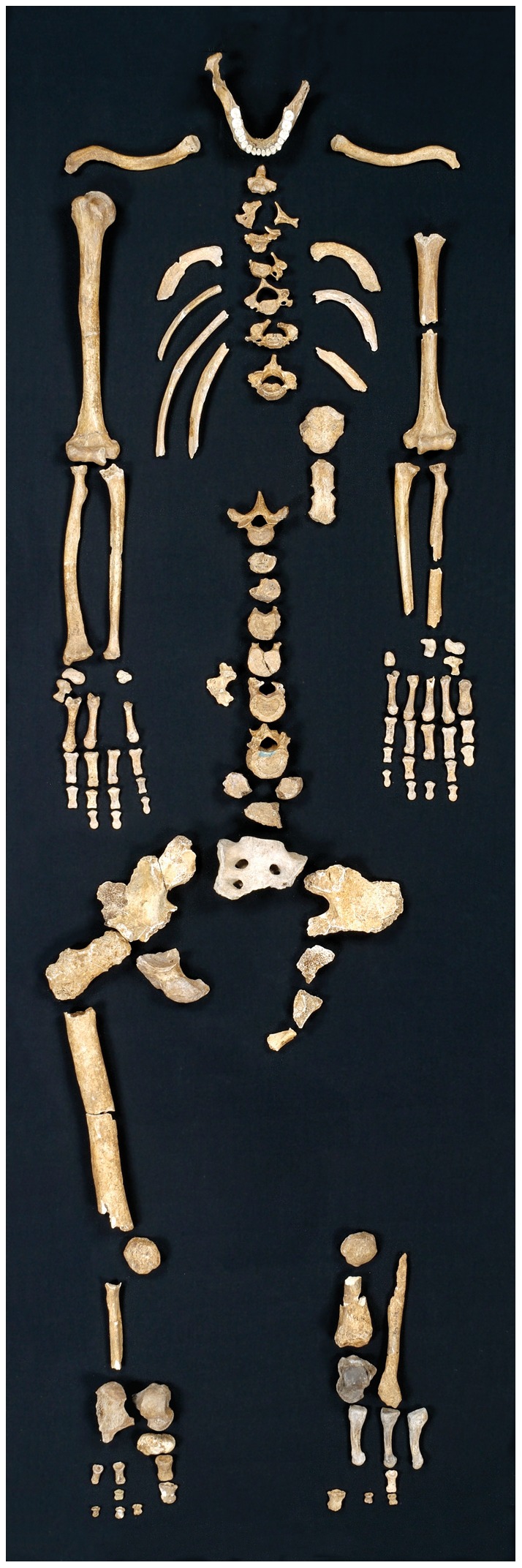
The skeleton of Regourdou 1. Photo credit: Collections Ville de Périgueux, Musée d'Art et d'Archéologie du Périgord: Inv. 85.3.

Regourdou 1 was discovered in 1957 in a collapsed rock shelter near Lascaux in the Dordogne of southwestern France. In the deposits were remains of a partial skeleton (Figure 1) along with fragmentary pedal remains of a second individual [18], [19]. Associated with La Quina type Mousterian [20] and considered to be from OIS 4, it is one of the oldest Neandertal skeletons from Western Europe [21], [22]. The skeleton (Regourdou 1) was likely part of a burial [18], [21], but the bones were disordered and incomplete, due to taphonomic conditions and early excavation procedures. Regourdou 1 consists of mandible and primarily the parts of the upper torso with scattered vertebra, the upper 1/2 of the sacrum, elements from the right and left ilia, and some bones of the lower limb, notably for the right side [23], [24]. Based on mandibular tooth wear and the closed medial clavicle epiphyses Regourdou 1 was probably between 23–30 years old at death, using modern epiphyseal closure data [25]. Sex is generally considered ‘indeterminate’ based on the small size of the long bones [19], [24], but the anatomical proportions of the body and alae in the superior sacrum [26], [27] suggest that it was a male. Despite the small size of the posterior teeth [20], Regourdou 1′s canine breadth is large and at 10.0 mm it is greater than all European Würm Neandertal females (mean  = 8.2 mm, range: 7.5–9.7 mm, n = 10) and at the top of the range for Neandertal males (mean  = 9.5 mm, range: 8.8–10.1 mm, n = 12). Like Froehle and Churchill [28], we suspect Regourdou 1 was a male but this is irrelevant to our analysis.

**Figure 2:**
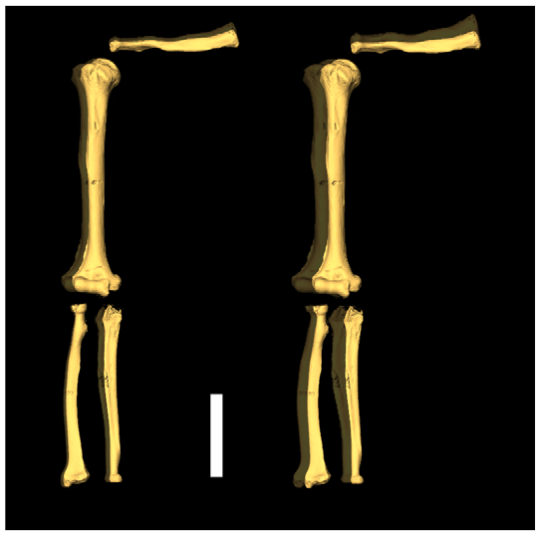
Microtomographic-based 3D reconstruction of the right upper limb chain of Regourdou 1 (in anterior view) showing the average degree of bilateral asymmetry (in %) assessed for the cortical area (CA, left) and the polar second moment of area (J, right) and rendered by virtual superimposition (semi-transparency, in gray) to the original outline of each element (in gold). To emphasize differences among the different elements, the amount of original asymmetry has been doubled. Scale bar is 10 cm.

## Results: Handedness from the Skeletal Elements

Vandermeersch and Trinkaus [19] were the first to document left/right asymmetry in the upper limb skeleton. They found that the right side was metrically larger and showed overall greater cross-sectional diameters than the left, which they attributed to right-handedness. For seven metrics of the humerus, radius and ulna, the right side is between 5–13% larger than the left, leading Vandermeersch and Trinkaus [19, p. 473] to conclude … “if upper limb asymmetry can be correlated with handedness, Régourdou 1 was right-handed.”

**Figure 3:**
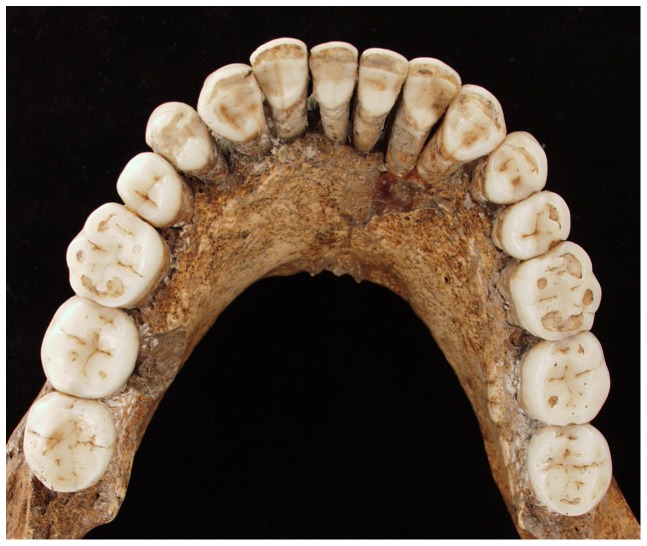
Occlusal view of Regourdou 1 mandible. The apparent malpositioning of the teeth is due to reconstruction. Photo credit: P Sémal, Royal Belgian Institute of Natural Sciences, Brussels.

**Figure 4:**
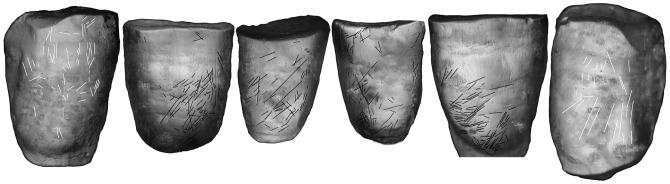
Labial scratches on Regourdou 1′s anterior teeth.

**Figure 5:**
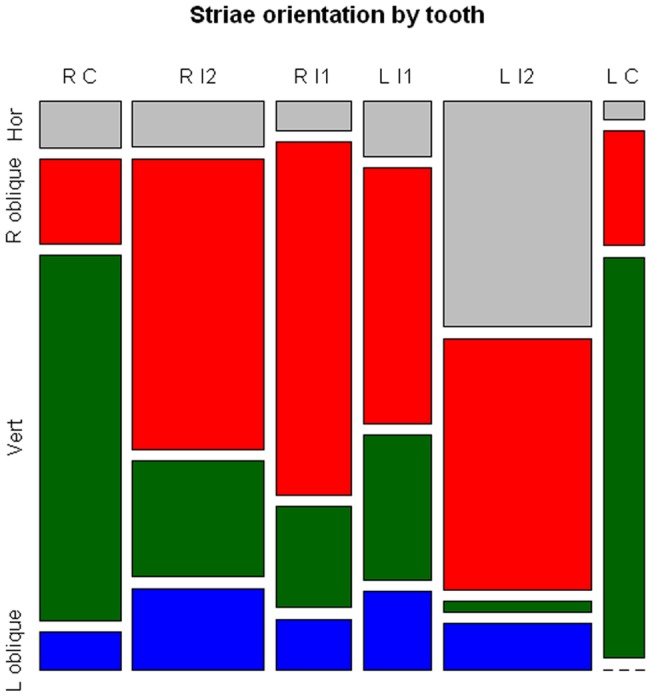
Mosaic plot of scratch angles on all anterior teeth. Scratch intervals follow ref 7.

**Figure 6:**
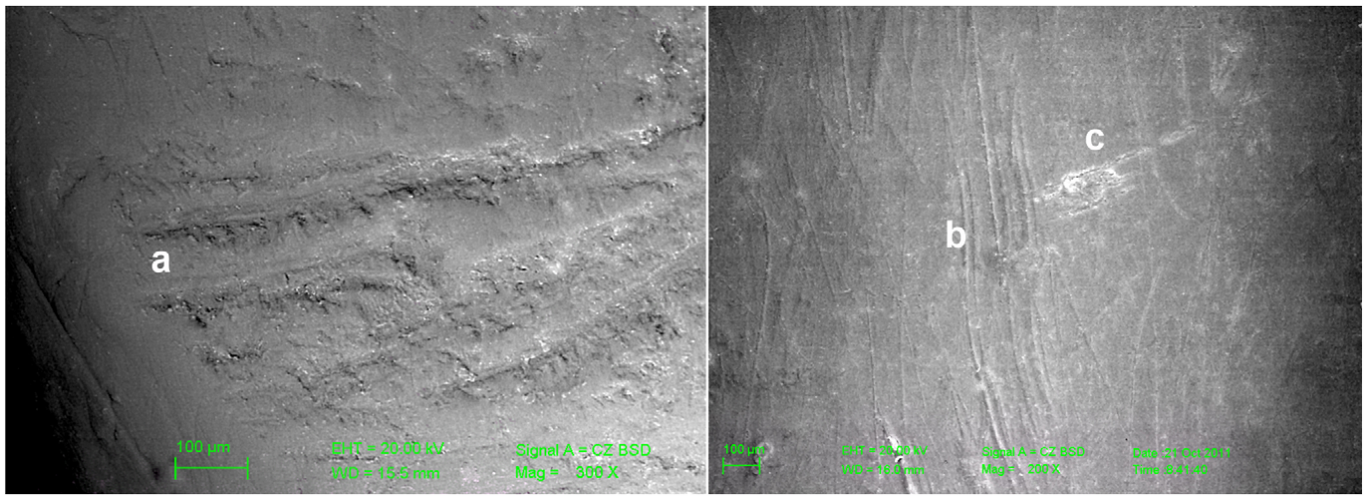
SEMs of striations on left I_2_ and right C. Left: Lower medial portion of the labial aspect of the left second incisor of Regourdou 1 (left) showing (a) some V-shaped, relatively thick sub-horizontal scratches associated with a few thinner secondary striae occurring within the major striae. The medial margin of the tooth is on the left. Right: Central portion of the labial aspect of the right canine showing a network of mostly sub-vertical thin scratches (b), which we identify as an occlusal scratch. (c) is an abrasion spot associated with a major oblique scratch of taphonomic origin, most likely from a sediment particle. Scale bar is 100 µm.

Following a preliminary biomechanical analysis of the humeri [29], synchrotron radiation microtomographic images were used to further document right/left endostructural and volumetric (cortical thickness-related) differences for the clavicle, humerus, radius and ulna. This represents the first integrated analysis concerning the entire upper limb skeletal chain (excluding bones of the hand) of a Neandertal (29]. Comparative variation across the shaft in cross-sectional geometric properties virtually assessed at three standardized sites (see Methods, Text S1) and three-dimensional cortical bone distribution were done. Variation in cortical area (CA), which relates to the resistance of the shaft to compressive/tensional axial loads, and polar second moment of area (J), reflecting the torsional and average bending rigidity [31], shows an ipslateral asymmetry for all four bones. The entire set of results supports a systematically higher biomechanical resistance to charges in compression/tension and, mostly, in torsion characterizing the right upper limb of this Neandertal individual. Interestingly, the microtomographic-based record shows that, despite the functional unity of the upper limb chain, its segments possess individually, at different degrees, asymmetry in strength loads (Figure 2). The clavicle recorded the highest level of asymmetry (mean difference in CA  = 17.8%; J = 40.3%), closely followed by the ulna (16.2% and 38.5%, respectively); the radius for CA (8.4%) and the humerus for J (21%) are the least asymmetric shafts (see Table S1). Nonetheless, compared to the currently available Neandertal figures, which are mostly radiographically-based [32], [33], the humerus displays only a relatively modest degree of right dominance (mean difference in CA  = 11.7%; J = 21%). At the humeral midshaft, the Neandertal ranges of variation are 18.6–43.2% and 53.0–83.9% for CA and J, respectively, while their corresponding values in Regourdou 1 (measured at 44%, as the midshaft is lacking on the left humerus) correspond to 11% and 16.1%.

Along with cross-sectional geometric properties, the same pattern of fluctuating bilateral asymmetry in Regourdou 1 is shown when cortical bone volume distribution is considered. In all but the humerus, asymmetry is expressed to a greater extent at the distal (lateral for the clavicle) portion of the shaft (25–45% of the biomechanical length for all but the humerus, where it corresponds to the 24–44% segment). For this portion, volumetric asymmetry ranges from 8.3% in the humerus (its proximal portion reaching 15.6%), up to 23.1% in the clavicle (see Tables S1 & S2).

As a whole, while the extent of bilateral asymmetry displayed by its humerus was not as marked as recorded in other Neandertal individuals, this new body of data shows that Regourdou 1 was consistently larger on the right side for most of the external and endostructural measures. From the asymmetry in the upper limb bones, Regourdou 1 was right-handed.

## Results: The Mandibular Teeth

Regourdou preserves a complete set of fully erupted, permanent teeth (Figure 3). All show little or moderate attrition, but occlusal wear on the incisors and canines is more marked than on the premolars and molars, a feature typical of Neandertals. Using Molnar's scale the anterior teeth show wear stages 4–5, while premolar and molar wear ranges is less, ranging between 2–4 [34]. This differential wear is an indication that Neandertals used their anterior teeth as a ‘third hand’ in manipulating objects [35]. Often called the ‘stuff and cut’ technique [36], likely more than food was involved. Heavier occlusal wear on the anterior teeth is matched by marked scratching on the labial (lip) surfaces of the incisors and canines. Unaffected by this wear, the labial aspect of all teeth is well-preserved, without evidence of much post-depositional corrosion or abrasion.

Figure 4 shows the heavily scratched labial surfaces of the six mandibular teeth of Regourdou 1. Striae are similarly distributed between the right (52.8%) and left (47.2%) sides, but not across the six teeth (Figures S1, S2 and S3). About 78% (297/382) of the scratches occur on the incisors with the remainder on the two canines. Together, the four incisors average 77.7 scratches and lateral incisors show nearly twice the number of scratches (mean  = 98) compared to the central incisors (mean  = 50). Striae are almost evenly distributed on the labial aspect of booth central incisor. Conversely, in both lateral incisors, the striations are concentrated on the mesial portion of the labial aspect, close to the neck. This evidence supports the interpretation of striae produced by the occasional hit of a stone tool across the labial tooth face. Finally, the striae on both lateral incisors are deeper and superimposed, indicating they were made at different times. The canines carry fewer scratches with more on the right (n = 57) than the left (n = 28). Moreover, the scratches on both canines tend to be shorter, thinner and less expressed than the scratches on the incisors.

Scratches were quantified using the intervals first proposed by Bermúdez de Castro et al. [7]. Appearing exclusively on the labial face of canines and incisors, 382 scratches were scored into these teeth. Right oblique marks (>22.5°−67.5° %) were the most common (45.0%) and left oblique (>112.5°–157.5°) the least common (10.2%) for all teeth. Vertical striations (>67.5°–112.5°) accounted for 27.5% and horizontal striations (0°–22.5°, >157.5°–180°) for the remainder (17.3%). We illustrate the distribution of the marks by mosaic plots (Figure 5), where percentages of the four intervals are given for each tooth (see Data Files S1, S2, S3, S4, S5 and S6). The dominant pattern for all four incisors is right oblique ranging between 47.1% and 66.0% of all marks. These are all significantly different from an equal probability of occurring at the >.001 level with chi^2^ (Table S3). While each incisor shows an array of angled marks, in these teeth the “right oblique category” consistently has the highest incidence of the four intervals. At least for the four lower incisors, the pattern shows consistent right-hand manipulations. For the incisors, the right oblique pattern is the most common (48.1%) and the left oblique the least common (10.2%), with the other categories intermediate.

Unlike all other specimens we have analyzed [5], [9], the canines preserve a different pattern, indicating that another action was performed on these teeth. In both canines vertical scratches are the most common, representing 68.4%–75.0% of the striations. In these cases right oblique marks are the next most common orientation (15.8%–21.4%), but these are far less frequent than the vertical marks. We suspect these scratches are related to mastication, lending no information to handedness. They do not contradict the consistent right-hand pattern on the four incisors, since in both canines left-handed scratches are rare (0%–7.0%).

The labial aspect of the Regourdou 1 second left mandibular incisor shows deep and relatively thick sub-horizontal micro-traces clustered towards its mesial inferior region. As revealed by both stereo-microscopic and SEM analyses (Fig. 6a), their typical V-shaped outline and the presence of secondary striae occurring within the major microfeatures are indicative of the repeated passage of a hard object, likely a lithic tool across the labial face. Compared to cutmarks produced by stone tools on bone [9], [39]–[40], striations on the teeth tend to be fainter, with fewer sharp, secondary striae. Unlike the cutmarks on bone, which are associated with butchering dead animals, these enamel striations occur in the living individual with the oral environment and saliva having a smoothing effect on the marks over the lifetime of the individual. At other sites like Krapina [5] and Sima de los Huesos [17], labial scratches occur in newly erupted permanent and in deciduous anterior teeth, indicating the cutting activity begins early in life, which accounts for the overstriking and smoothing of marks.

In contrast to the pattern characterizing the lower incisors, the SEM image of the labial aspect of the right canine shows a group of mostly vertically-oriented relatively thin, short scratches with the longest reaching ca. 1 mm length (Fig. 6b), The presence of an abrasion spot associated with a major scratch that runs obliquely mesio-distally and partially overprints the sub-vertical labial network likely results from a post-depositional (taphonomic) action by sedimentary particles. We have not seen this pattern of vertical striations in other Neandertal canines.

## Conclusions

Regourdou 1 joins 16 of 18 European Neandertals (88.9%) who were clearly right–handed, along with their likely ancestors from Sima de los Huesos, all of whom were right-handed [9], [17]. This predominant right-handed frequency (∼90.0%) has implications beyond simple hand preference, since handedness is a uniquely human trait [41], [42] with right-handers outnumbering left-handers in all world-wide cultures occurring in approximately a 9∶1 ratio [43], [44]. Based on fetal behavior (e.g. thumb sucking) handedness asymmetry begins in utero [45]–[47] by as early as the tenth fetal week [48] and correlates with adolescent handedness in the same individuals [46]. It also is linked to further brain structural asymmetries developing before language ability [49], indicating a genetic underpinning to handedness [50], [51]. Fetal behavioral evidence of asymmetry long presages later skeletal expression of right/left differences, which begins after one year and affects different dimensions of the humerus discordantly [52].

The long known connection among brain asymmetry, handedness and language in living populations serves as a proxy for estimating brain lateralization in the fossil record [9], [17] and the likelihood of language capacity in fossils. That is, if Neandertal handedness asymmetry resembled the more ambidextrous ape frequencies, the fossils would be judged incompletely lateralized. But just to the contrary Neandertals appear to be highly lateralized like modern humans. The concordance of arm and dental evidence for handedness in Regourdou links activities directed by asymmetrical arm movements with the traces of the activities preserved on the teeth. These observations are completely concordant with earlier work showing Neandertals are lateralized like modern *Homo sapiens*.

These observations, coupled with evidence from the archaeological record [53], paleoneurology [54], audition [55], [56], modern hyoid morphology and placement [57], [58], Neandertal DNA with the modern FOXP2 variant [59] and complex/ritual behavior [60]–[68] extend evidence for language competence deep into the European past.

## Supporting Information

Figure S1 **Correlations of scratch length and angle for each tooth.** Low correlation coefficients (−0.03–0.07) indicate there is no relation between scratch angle and scratch length.
(TIFF)

Figure S2 **Frequency distributions of all scratches per tooth at 10° intervals.** Incisors show predominantly scratches below 90°. Both canines show the highest frequencies of scratches around 90°.
(PDF)

Figure S3 **Box plot of the total number of scratches for each Regourdou 1 anterior tooth, using intervals in** [**69**] **.**
(TIFF)

Data File S1 **Raw data in Microsoft Xls format for dental scratch analysis on left canine.**
(XLS)

Data File S2 **Raw data in Microsoft Xls format for dental scratch analysis on right canine.**
(XLS)

Data File S3 **Raw data in Microsoft Xls format for dental scratch analysis on left first incisor.**
(XLS)

Data File S4 **Raw data in Microsoft Xls format for dental scratch analysis on right first incisor.**
(XLS)

Data File S5 **Raw data in Microsoft Xls format for dental scratch analysis on left second incisor.**
(XLS)

Data File S6 **Raw data in Microsoft Xls format for dental scratch analysis on right second incisor.**
(XLS)

Text S1 **Methods.**
(DOC)

Table S1 **Degree of bilateral asymmetry (%) in Regourdou 1 for the cortical area (CA) and the polar second moment of area (J) measured at three cross-sectional levels (distal / lateral, around the midshaft, proximal / medial) of the diaphysis on the clavicle (35%, 50%, 65%), the humerus (35%, 44%, 65%), the radius (35%, 45%, 65%), and the ulna (35%, 50%, 65%).**
(DOC)

Table S2 **Degree of bilateral asymmetry (%) in Regourdou 1 for the cortical bone volume (CV) distinctly assessed for the distal (dCV, lateral for the clavicle) and the proximal (pCV, medial for the clavicle) portions of the diaphysis on the clavicle (dCV: 25**–**45%; pCV: 60**–**80%), the humerus (dCV: 24**–**44%; pCV: 60**–**80%), the radius (dCV: 25**–**45%; pCV: 61**–**80%), and the ulna (dCV: 25**–**45%; pCV: 60–80%).**
(DOCX)

Table S3 **Summary straie statistics for all Regourdou 1 teeth (intervals according to** [**69**] **).**
(DOCX)

## References

[1] Henri-Martin H (1923) Les hommes fossiles de la Quina. Paris: Doin. 260p.

[2] Koby FE (1956) Une incisive néandertalienne trouveé en Suisse. Ver Natur Ges Basel 67: 1–15.

[3] Patte E (1960) Découverte d'un Néandertalien dans la Vienne. L'Anthropol 64: 512–517.

[4] de Lumley MA (1973) Anténéandertaliens et Néandertaliens du basin méditerranéen occidental Européen. Étude Quat 2: 1–626.

[5] Lalueza C, Frayer DW (1997) Non-dietary marks in the anterior dentition of the Krapina Neanderthals. Int J Osteoarch 7: 133–149.

[6] Frayer DW, Lalueza-Fox C, Fiore I, Radovčić J, Bondioli L (2010) Right-handed Neandertals: Vindija and beyond. J Anthrop Sci 88: 113–127. 20834053

[7] Bermúdez de Castro JM, Bromage TG, Fernández-Jalvo Y (1988) Buccal striations on fossil human anterior teeth: evidence of handedness in the middle and early Upper Pleistocene. J Hum Evol 17: 403–412.

[8] Lozano M, Bermúdez de Castro, JM Carbonell, E Arsuaga (2008) JL (2008) Non-masticatory uses of anterior teeth of Sima de los Huesos individuals Sierra de Atapuerca, Spain. J Hum Evol 55: 713–728. 18617220 10.1016/j.jhevol.2008.04.007

[9] Frayer DW, Lozano M, Bermúdez de Castro JM, Carbonell E, Arsuaga JL, et al (2011) More than 500,000 years of right-handedness in Europe. Laterality 17: 51–69. 21500084 10.1080/1357650X.2010.529451

[10] Puech PF, Puech S, Cianfarani F, Albertini H (1987) Tooth wear and dexterity in *Homo erectus*. In: Giacobini G, editor. Hominidae. Milan: JACA, 247–250.

[11] Hardyck C, Goldman R, Petrinovich L (1975) Handedness and sex, race and age. Hum Biol 47: 369–375. 1176108

[12] Percelle IB, Ehrman L (1994) An international study of human handedness: The data. Behav Gen 24: 217–227. 10.1007/BF01067189 7945152

[13] Cashmore L, Uomini N, Chapelain A (2008) The evolution of handedness in humans and great apes: A review and current issues. J Anthrop Sci 8: 7–35. 19934467

[14] Lozano M, Bermúdez de Castro JM, Martinón-Torres M, Sarmiento S (2004) Cutmarks on fossil human anterior teeth of the Sima de los Huesos site (Atapuerca, Spain). J Archaeol Sci 31: 1127–1135.

[15] Bax J, Ungar P (1999) Incisor labial surface wear striations in modern humans and their implications for handedness in Middle and Late Pleistocene hominids. Int J Osteoarch 9: 189–198.

[16] Lalueza C (1992) Information obtained from a microscopic examination of cultural striations. Int J Osteoarch 2: 155–169.

[17] Lozano M, Mosquera M, Bermúdez de Castro JM, Arsuaga J, Carbonell E (2009) Right-handedness of *Homo heidelbergensis* from Sima de los Huesos Atapuerca, Spain 500,000 years ago. Evol Hum Behav 30: 369–376.

[18] Bonifay E, Vandermeersch B (1962) Dépôts rituels d'ossements d'ours dans le gisement moustérien de Régourdou (Montignac, Dordogne). C R Acad Sci 255D: 1635–1636.

[19] Vandermeersch B, Trinkaus E (1995) The postcranial remains of the Régourdou I Neandertal: the shoulder and arm remains. J Hum Evol 28: 439–476.

[20] Maureille B, Rougier H, Houët F, Vandermeersch B (2001) Les dents inferieures du néandertalien Régourdou 1 (site de Régourdou, commune de Montignac, Dordogne): analyses métrique et comparative. Paléo 13: 183–200.

[21] Turq A, Jaubert J, Maureille B, Laville D (2008) Le cas des sépultures Néandertaliennes du Sud-Ouest: et si on les vieillissait? In: Vandermeersch B, Cleyet-Merle J-J, Maureille B, Turq A, editors. Première Humanité, Gestes Funéraires des Néandertaliens. Paris: Réunion des Musées Nationaux, 40–41.

[22] Cavanhié N (2009/10) L'ours qui a vu l'homme? Étude archéozoologique et taphonomique du site paléolithique moyen de Regourdou (Montignac, Dordogne, France) Paleo 21: 39–63.

[23] Madelaine S, Maureille B, Cavanhié N, Couture-Veschambre C, Bonifay E, et al (2008) Nouveaux restes humains moustériens rapportés au squelette néandertalien de Regourdou 1 (Regourdou, commune de Montignac, Dordogne, France). Paleo 20: 101–114.

[24] Meyer V, Brůžek J, Couture C, Santos F, Dutailly B, et al (2011) Régourdou 1: A new Neandertalian pelvis, preliminary study and perspectives of functional interpretation. Amer J Phys Anthropol 144: 214.

[25] White TD, Folkens PA (2000) Human osteology. New York: Academic, 563p.

[26] Anderson JE (1972) The human skeleton. Ottawa: National Museum of Canada. 164p.

[27] Flander LB (1978) Univariate and multivariate methods for sexing the sacrum. Amer J Phys Anthropol 49: 103–110. 677289 10.1002/ajpa.1330490116

[28] Froehle AW, Churchill SE (2009) Energetic competition between Neandertals and anatomically modern humans. PaleoAnthropol 2009: 96–116.

[29] Volpato V, Couture C, Macchiarelli R, Vandermeersch B (2011) Endostructural characterisation of the Regourdou 1 Neanderthal proximal arm: bilateral asymmetry and handedness. In: Condemi S, Weniger G-C, editors. Continuity and discontinuity in the peopling of Europe: One hundred fifty years of Neanderthal study, Vertebrate Paleobiology and Paleoanthropology. New York: Springer, 175–178.

[30] Bayle P, Bondioli L, Macchiarelli R, Mazurier A, Puymerail L, et al. (2011) Three-dimensional imaging and quantitative characterization of human fossil remains. Examples from the NESPOS database. In: Macchiarelli R, Weniger G-C, editors. Pleistocene databases. Acquisition, storing, sharing. Mettmann: Wissenschaftliche Schriften des Neanderthal Museums 4, 29–46.

[31] Ruff CB (2008) Biomechanical analyses of archaeological human skeletal samples. In: Katzenberg MA, Saunders SR, editors. Biological anthropology of the human skeleton, 2nd ed. Hoboken: Wiley-Liss, 183–206.

[32] Trinkaus E, Churchill SE, Ruff CB (1994) Postcranial robusticity in *Homo.* II: Humeral bilateral asymmetry and bone plasticity. Amer J Phys Anthropol 93: 1–34. 8141238 10.1002/ajpa.1330930102

[33] Churchill SE, Formicola V (1997) A case of marked bilateral asymmetry in the upper limbs of an Upper Palaeolithic male from Barma Grande (Liguria), Italy. Int J Osteoarchaeol 7: 18–38.

[34] Molnar S (1971) Human tooth wear, tooth function, and cultural variability. Amer J Phys Anthropol 34: 175–190. 5572602 10.1002/ajpa.1330340204

[35] Wolpoff MH (1999) Paleoanthropology. New York: McGraw-Hill, 878p.

[36] Brace CL (1991) The stages of human evolution. Englewood Cliffs (NJ): Prentice-Hall. 200p.

[37] Shaw CN, Hofmann CL, Petraglia MD, Stock JT, Gottschal JS (2012) Neandertal humeri may reflect adaptation to scraping tasks, but not spear thrusting. PLoS One 7(7): e40349 doi:10.1371/journal.pone.0040349. 22815742 10.1371/journal.pone.0040349 PMC3399840

[38] Olsen LS, Shipman P (1988) Surface modification on bone: trampling versus butchery. J Archaeol Sci 15: 335–353.

[39] Lyman RL (1994) Vertebrate taphonomy. Cambridge manuals in archaeology, Cambridge: Cambridge University Press, 524 p.

[40] Fischer JW (1995) Bone surface modifications in zooarchaeology. J Archaeol Meth Theory 2: 7–68.

[41] McGrew WC, Marchant LF (1997) On the other hand: current issues in and metaanalysis of the behavioral laterality of hand function in nonhuman primates. Ybk Phys Anthropol 40: 201–232.

[42] Cashmore L (2009) Can hominin ‘handedness’ be accurately assessed? Ann Hum Biol 36: 624–641. 19626485 10.1080/03014460902956733

[43] McManus C (2009) The history and geography of human handedness. In: Sommer EC, Kahn R, editors. Language lateralization and psychosis. Cambridge: Cambridge University Press, 37–57.

[44] Chapelain AS, Hogervorst E, Mbonzo P, Hopkins WD (2011) Hand preferences for bimanual coordination in 77 bonobos (*Pan paniscus*): replication and extension. Int J Primatol 32: 491–510.

[45] Hepper PG, Shahidullah S, White R (1991) Handedness in the human fetus. Neuropsych 29: 1107–1111. 10.1016/0028-3932(91)90080-r 1775228

[46] Hepper PG, Wells DL, Lynch C (2005) Perinatal thumb sucking is related to postnatal handedness. Neuropsych 43: 313–315. 10.1016/j.neuropsychologia.2004.08.009 15707608

[47] Kasprian G, Langs G, Brugger PC, Bittner M, Weber M, et al (2011) The prenatal origin of hemispheric asymmetry: An in utero neuroimaging study. Cerebr Cortex 21: 1076–1083. 10.1093/cercor/bhq179 20851852

[48] Hepper PG, McCartney GR, Shannon EA (1998) Lateralised behaviour in first trimester human foetuses. Neuropsych 36: 531–534. 10.1016/s0028-3932(97)00156-5 9705063

[49] Dubois J, Hertz-Pannier L, Cachia A, Mangin JF, Le Behan D, et al (2009) Structural asymmetries in the infant language and sensori-motor networks. Cerebr Cortex 19: 414–425. 10.1093/cercor/bhn097 18562332

[50] McManus IC, Bryden MP (1992). The genetics of handedness, cerebral dominance, and lateralization. In: Rapin I, Segalowitz SJ, editors. Handbook of neuropsychology, vol. 6. New York: Elsevier Science, 116–144.

[51] Corballis M (1997) The genetics and evolution of handedness. Psych Rev 104: 714–727. 10.1037/0033-295x.104.4.714 9337630

[52] Blackburn A (2011) Bilateral asymmetry of the humerus during growth and development. Amer J Phys Anthropol 145: 639–646. 21702005 10.1002/ajpa.21555

[53] Uomini NT (2009) The prehistory of handedness: archaeological data and comparative ethology. J Hum Evol 57: 411–419. 19758680 10.1016/j.jhevol.2009.02.012

[54] Holloway R (1985) The poor brain of *Homo sapiens neanderthalensis*: See what you please. In: Delson E, editor. Ancestors: The hard evidence. New York: Alan R. Liss, 319–324.

[55] Martínez I, Rosa M, Arsuaga JL, Jarabo P, Quam R, et al (2004) Auditory capacities in Middle Pleistocene humans from the Sierra de Atapuerca in Spain. PNAS 101: 9976–9981. 15213327 10.1073/pnas.0403595101 PMC454200

[56] Martínez I, Quam R, Arsuaga JL Lorenzo C, Gràcia A, et al (2009) Approche paleontològiques de l'évolution du langage: Un état des lieux. L'Anth 113: 255–264.

[57] Martínez I, Arsuaga JL, Quam R, Carretero JM, Gràcia A, et al (2008) Human hyoid bones from the Middle Pleistocene site of the Sima de los Huesos (Sierra de Atapuerca, Spain). J Hum Evol 54: 118–124. 17804038 10.1016/j.jhevol.2007.07.006

[58] Frayer DW, Nicolay CW (2000) Fossil evidence for the origin of speech sounds. In: Wallin NL, Merker B, Brown S, editors. The origins of music. Cambridge: MIT Press, 217–234.

[59] Krause J, Lalueza-Fox C, Orlando L, Enard W, Green R, et al (2007) The derived FOXP2 variant of modern humans was shared with Neandertals. Curr Biol 17: 1908–1912. 17949978 10.1016/j.cub.2007.10.008

[60] Bednarik R (1992) Palaeart and archaeological myths. Camb Archaeol J 2: 27–57.

[61] Caron F, d'Errico F, Del Moral P, Santos F, Zilhaõ J (2011) The reality of Neandertal symbolic behavior at the Grotte du Renne, Arcy-sur-Cure, France. PLoS ONE 6(6): e21545 doi:10.1371/journal.pone.0021545. 21738702 10.1371/journal.pone.0021545 PMC3126825

[62] Roebroeks W, Sier MJ, Nelson TK, de Loecker D, Parés JM, et al (2012) Use of red ochre by earlier Neandertals. PNAS, USA 109: 1889–1894. 22308348 10.1073/pnas.1112261109 PMC3277516

[63] Hayden B (2012) Neandertal social structure. Oxf J Archaeol 31: 1–26.

[64] Frayer DW, Orschiedt J, Cook J, Russell MD, Radovčić J (2006) Krapina 3: Cut marks and ritual behavior? Periodicum biologorum 108: 519–524.

[65] Zilhão J, Angelucci DE, Badal-García E, d'Errico F, Daniel F, et al (2010) Symbolic use of marine shells and mineral pigments by Iberian Neanderthals. PNAS 107: 1023–1028. 20080653 10.1073/pnas.0914088107 PMC2824307

[66] Peresani M, Fiore I, Gala M, Romandini M, Tagliacozzo A (2011) Late Neandertals and the intentional removal of feathers as evidenced from bird bone taphonomy at Fumane Cave 44 ky B.P., Italy. PNAS 108: 3888–3893. 21368129 10.1073/pnas.1016212108 PMC3054018

[67] Morin E, Laroulandie V (2012) Presumed symbolic use of diurnal raptors by Neanderthals. PLoS ONE 7(3): e32856 doi:10.1371/journal.pone.0032856. 22403717 10.1371/journal.pone.0032856 PMC3293908

[68] Lazuén T (2012) European Neanderthal stone hunting weapons reveal complex behaviour long before the appearance of modern humans. J Archaeol Sci 39: 2304–2311.

[69] Bermúdez de Castro JM, Bromage TG, Fernández-Jalvo Y (1988) Buccal striations on fossil human anterior teeth: evidence of handedness in the middle and early Upper Pleistocene. J Hum Evol 17: 403–412.

