# Supplementary figures and images for: Hand to Mouth in a Neandertal: Right-Handedness in Regourdou 1
Source: PLoS One. 2012 Aug 22;7(8):e43949. doi: 10.1371/journal.pone.0043949 (PMC3425541; doi:10.1371/journal.pone.0043949)

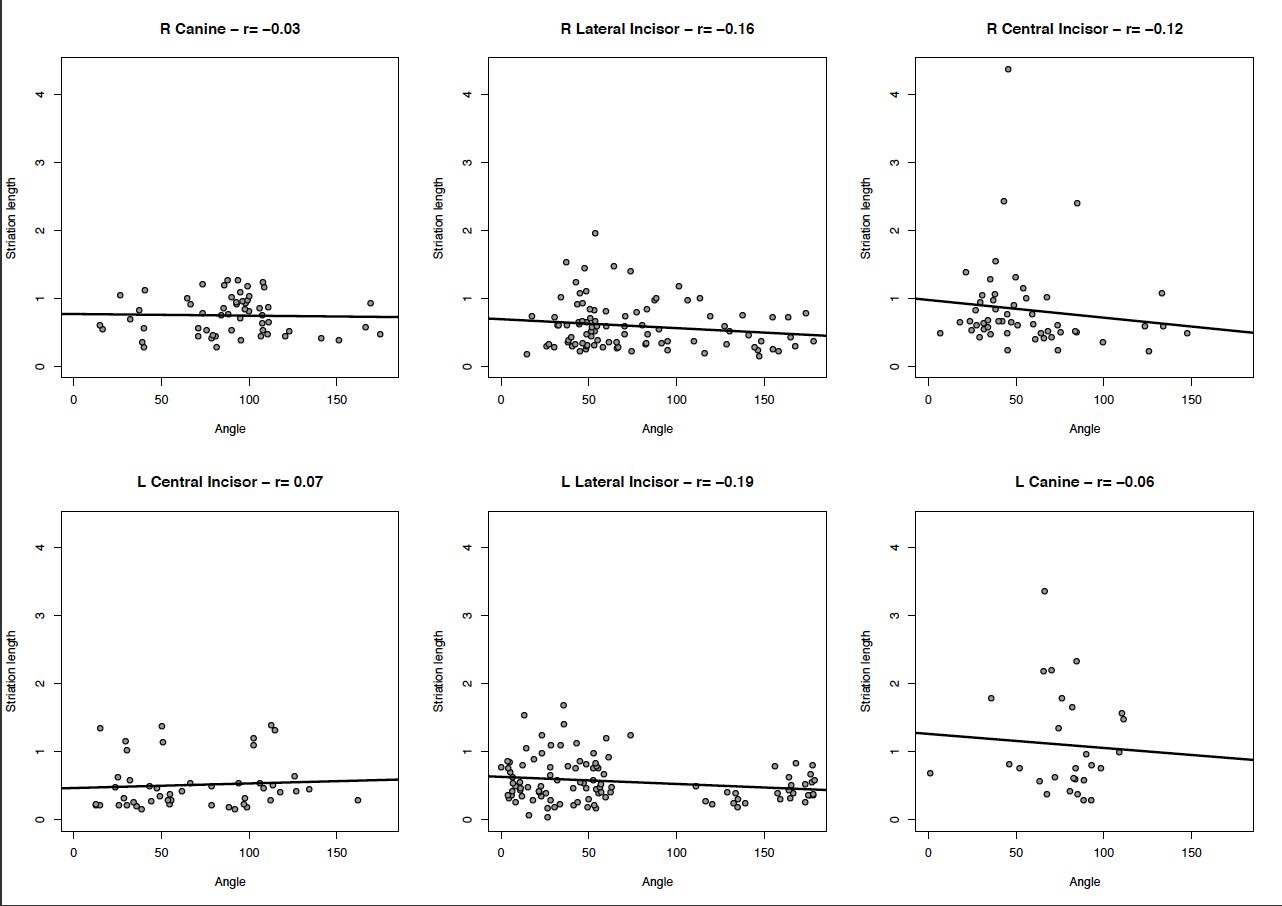

Supplement: Figure S1 — Correlations of scratch length and angle for each tooth. Low correlation coefficients (−0.03–0.07) indicate there is no relation between scratch angle and scratch length. (TIFF) [file pone.0043949.s001.tiff]

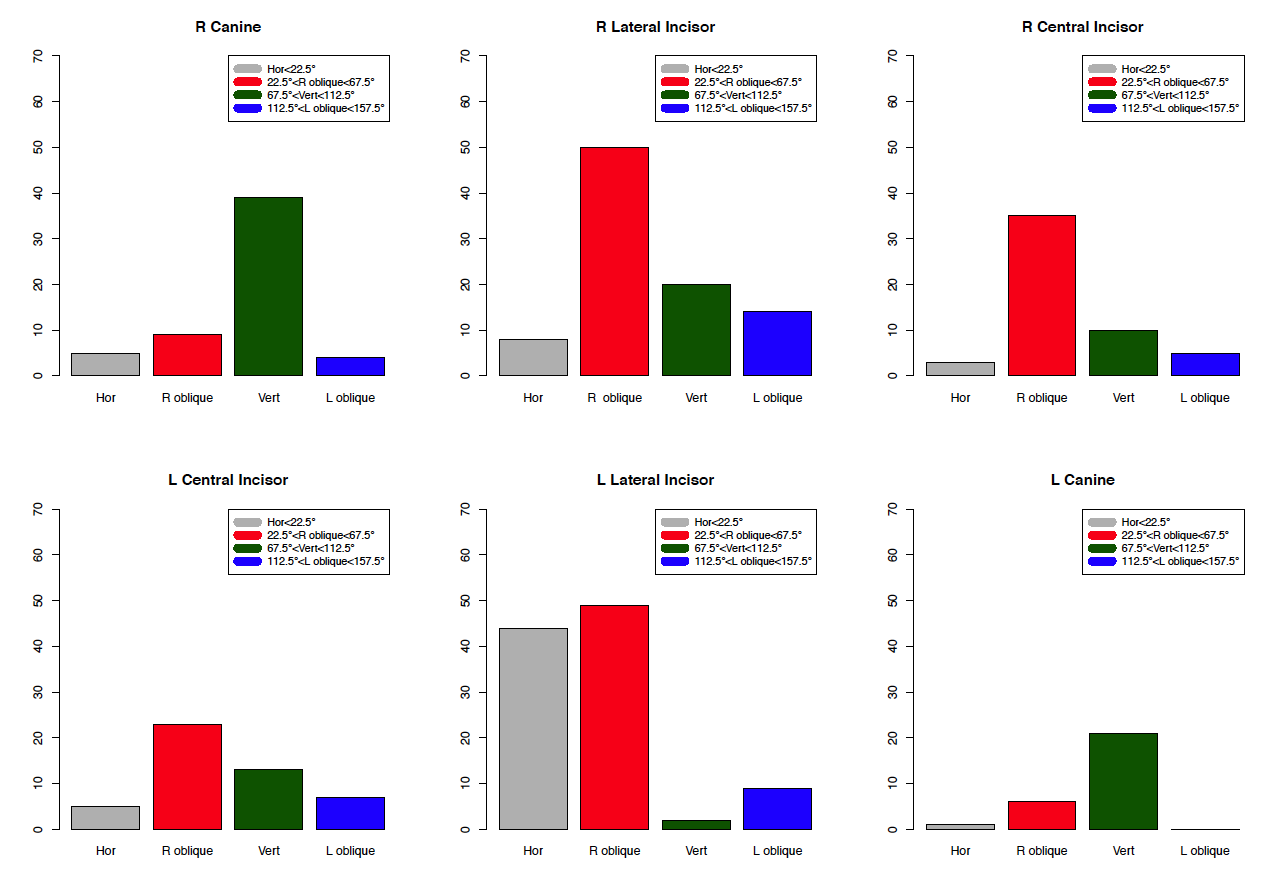

Supplement: Figure S3 — Box plot of the total number of scratches for each Regourdou 1 anterior tooth, using intervals in [69] . (TIFF) [file pone.0043949.s003.tiff]
