# Supplementary material for: Hand to Mouth in a Neandertal: Right-Handedness in Regourdou 1
Source: PLoS One. 2012 Aug 22;7(8):e43949. doi: 10.1371/journal.pone.0043949 (PMC3425541; doi:10.1371/journal.pone.0043949)
Supplement: Figure S2 — Frequency distributions of all scratches per tooth at 10° intervals. Incisors show predominantly scratches below 90°. Both canines show the highest frequencies of scratches around 90°. (PDF) [file pone.0043949.s002.pdf]

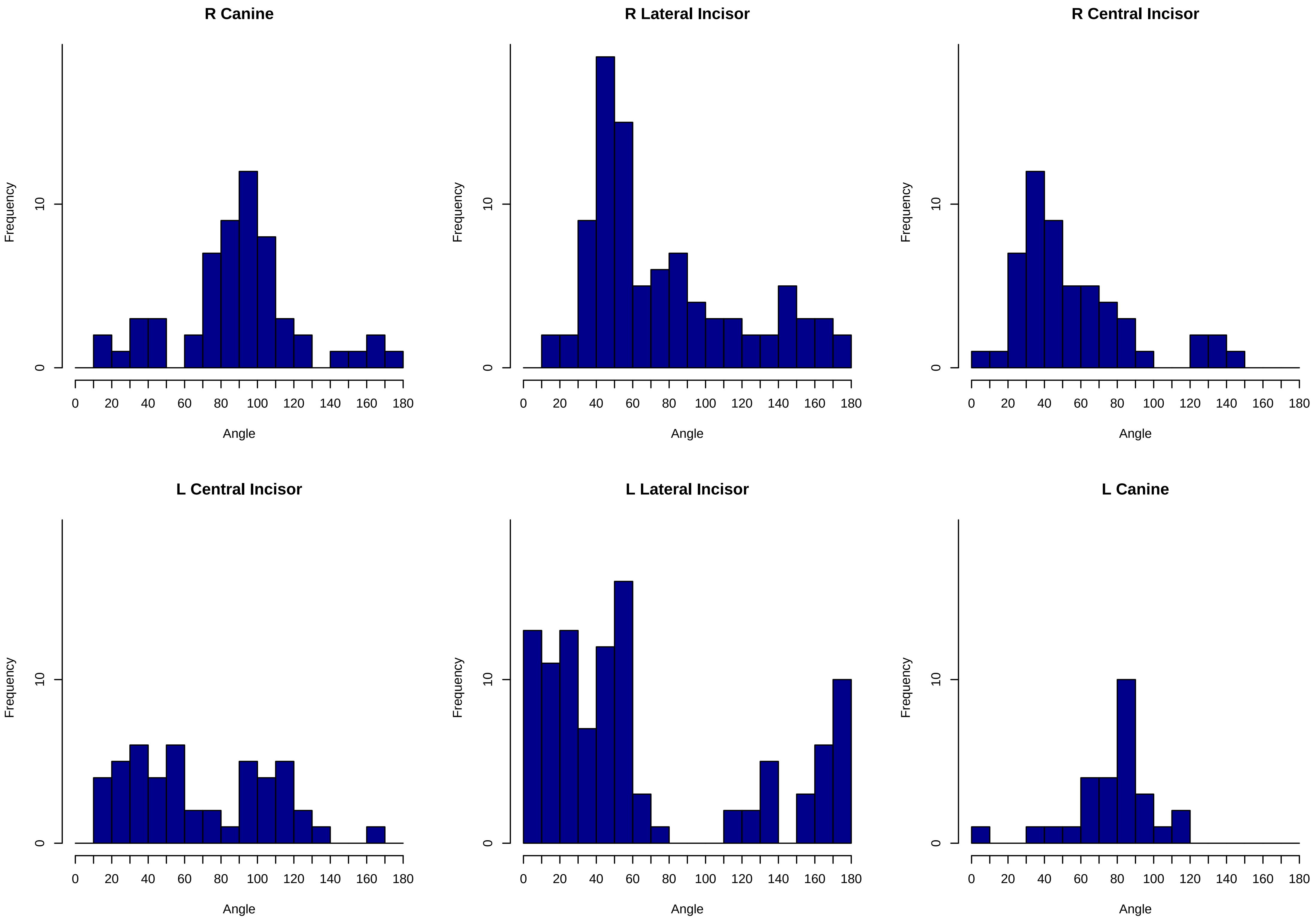
R Canine
R Lateral Incisor
R Central Incisor
L Central Incisor
L Lateral Incisor
L Canine
Frequency
0
10
Angle
0 20 40 60 80 100 120 140 160 180

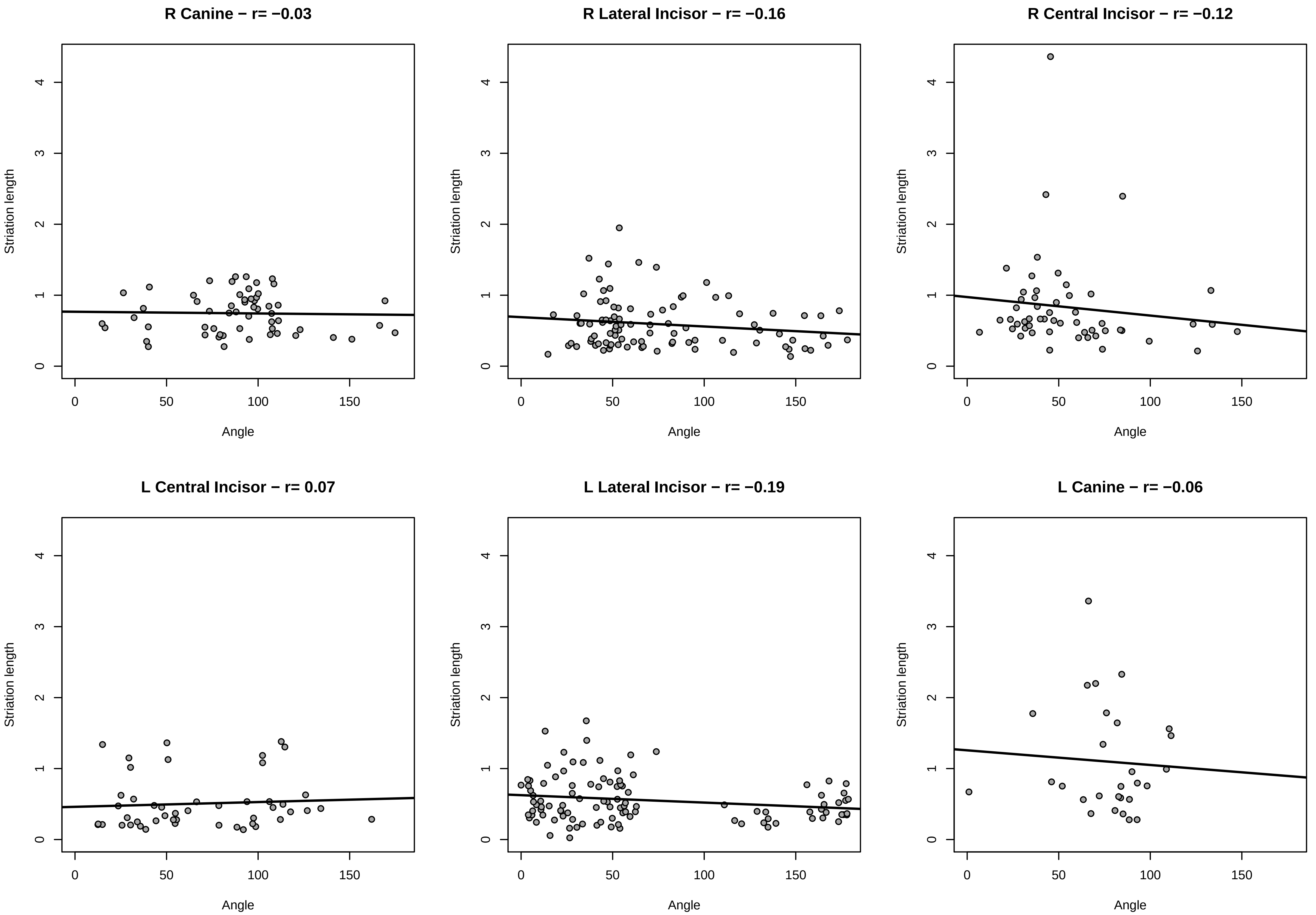
R Canine − r= −0.03
R Lateral Incisor − r= −0.16
R Central Incisor − r= −0.12
L Central Incisor − r= 0.07
L Lateral Incisor − r= −0.19
L Canine − r= −0.06
Striation length
Angle
0
1
2
3
4
50
100
150

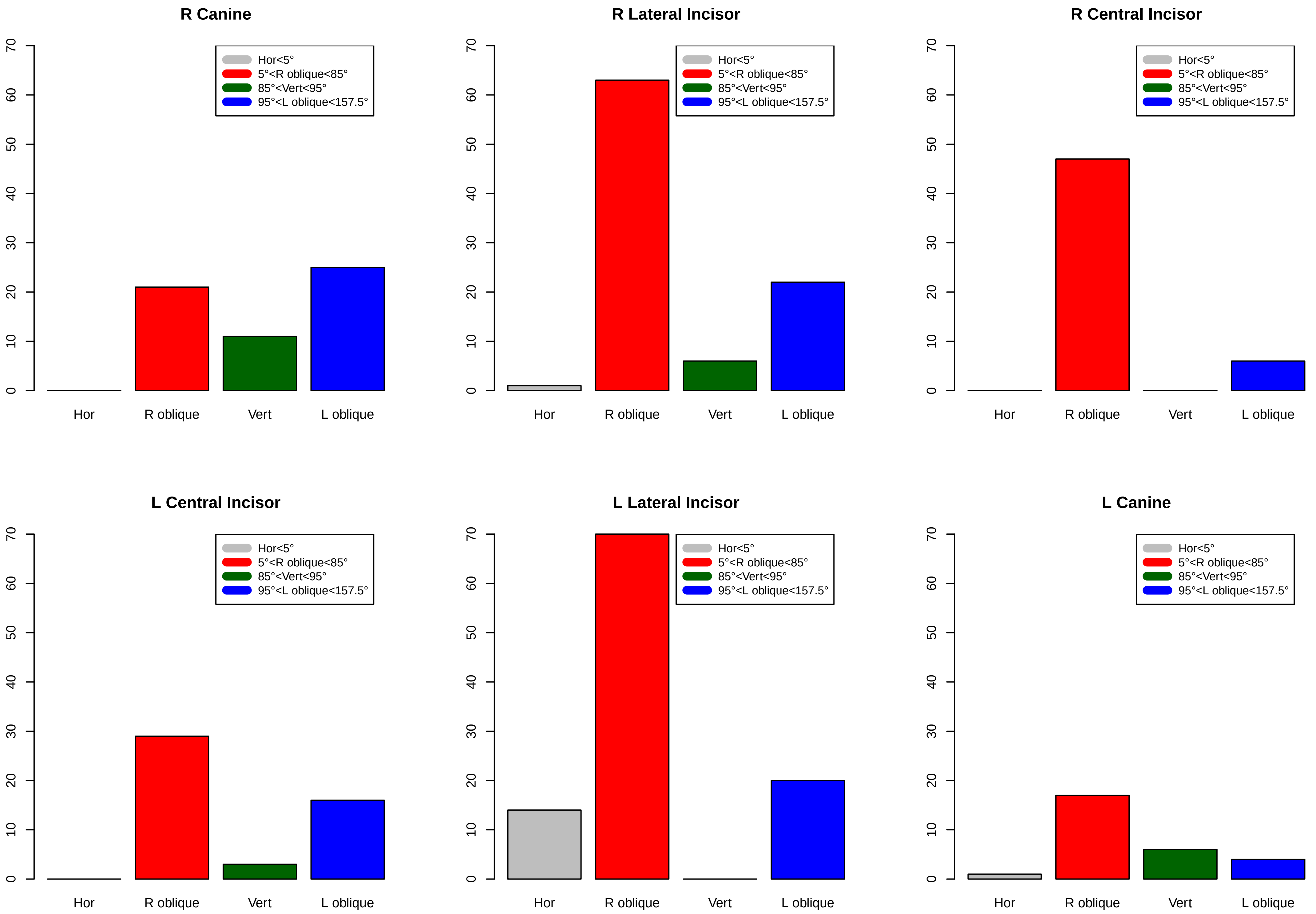
R Canine
R Lateral Incisor
R Central Incisor
L Central Incisor
L Lateral Incisor
L Canine
0
10
20
30
40
50
60
70
Hor<5°
5°<R oblique<85°
85°<Vert<95°
95°<L oblique<157.5°
Hor
R oblique
Vert
L oblique

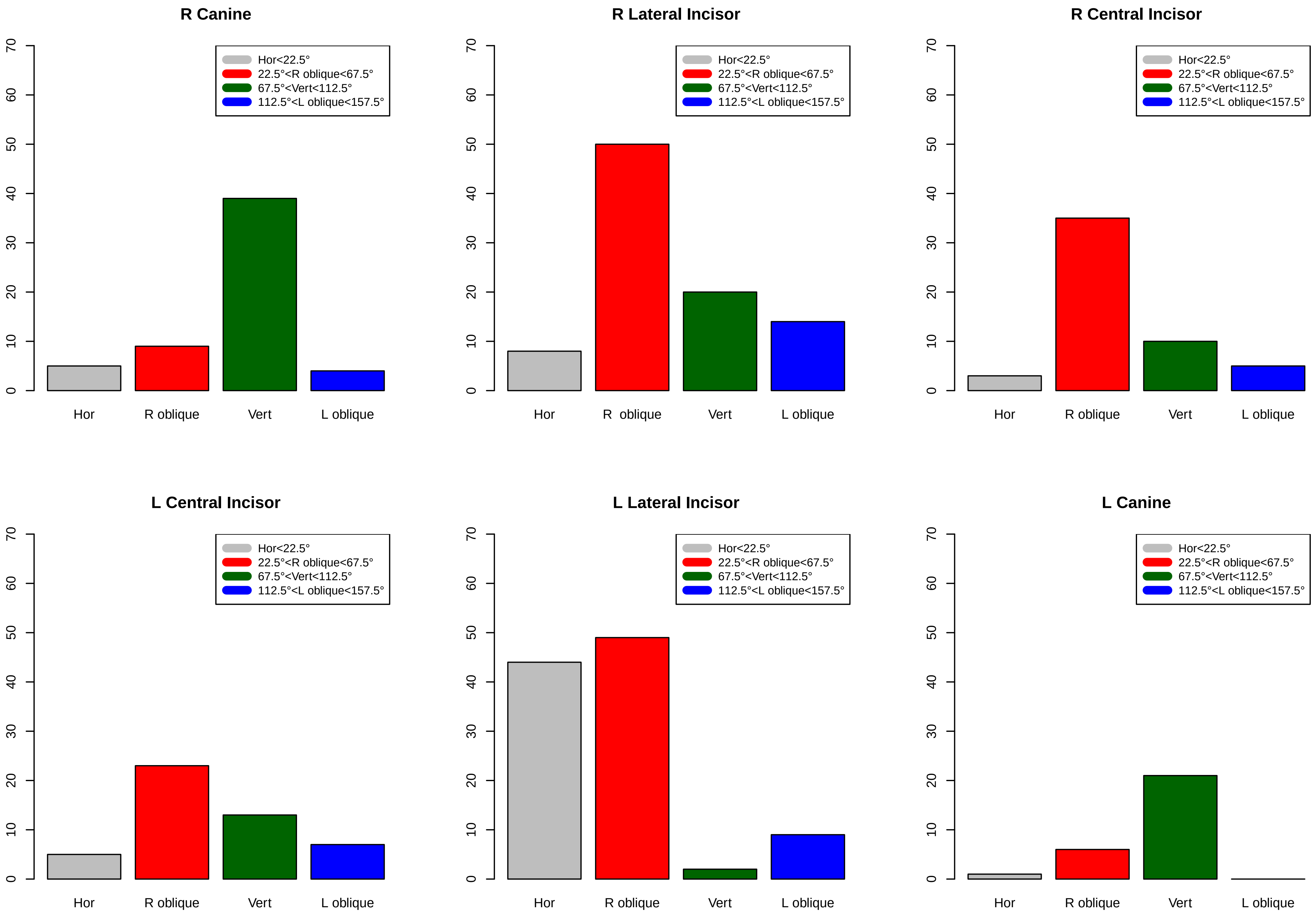
R Canine
R Lateral Incisor
R Central Incisor
L Central Incisor
L Lateral Incisor
L Canine
0
10
20
30
40
50
60
70
Hor<22.5°
22.5°<R oblique<67.5°
67.5°<Vert<112.5°
112.5°<L oblique<157.5°
Hor
R oblique
Vert
L oblique

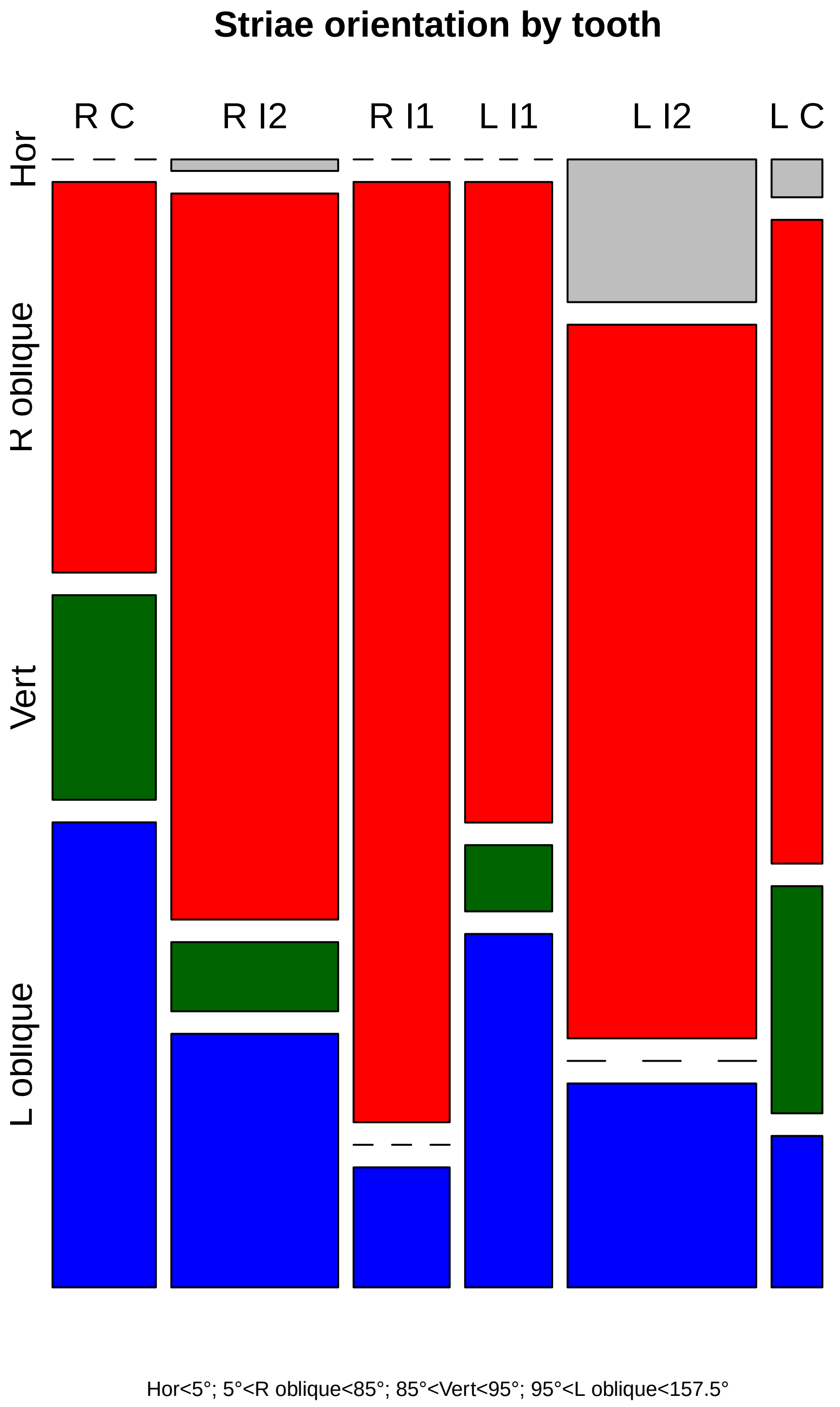
Striae orientation by tooth
R C
R I2
R I1
L I1
L I2
L C
Hor
R oblique
Vert
L oblique
Hor<5°; 5°<R oblique<85°; 85°<Vert<95°; 95°<L oblique<157.5°

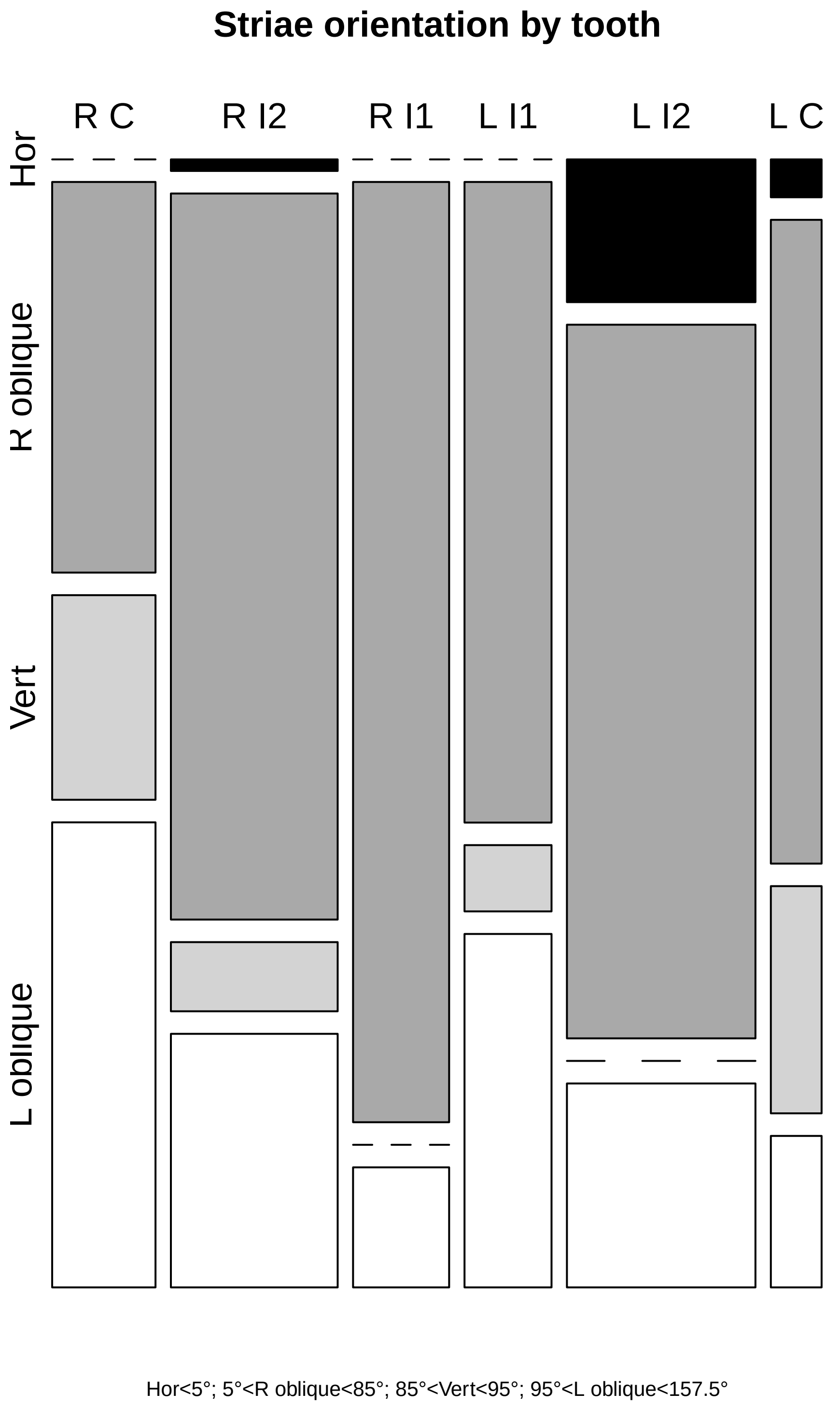
Striae orientation by tooth
R C
R I2
R I1
L I1
L I2
L C
Hor
R oblique
Vert
L oblique
Hor<5°; 5°<R oblique<85°; 85°<Vert<95°; 95°<L oblique<157.5°

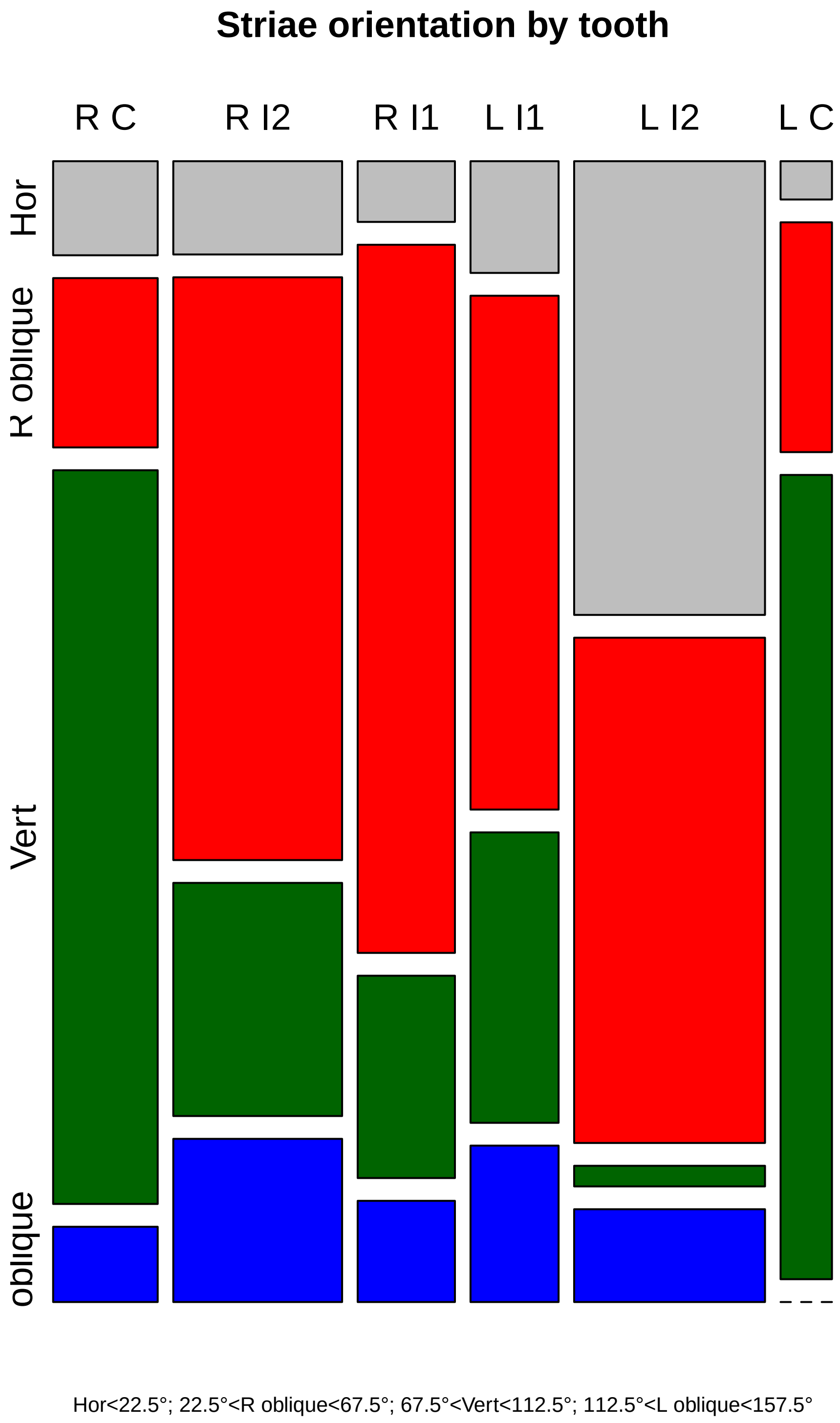
Striae orientation by tooth
R C
R I2
R I1
L I1
L I2
L C
Hor
R oblique
Vert
oblique
Hor<22.5°; 22.5°<R oblique<67.5°; 67.5°<Vert<112.5°; 112.5°<L oblique<157.5°

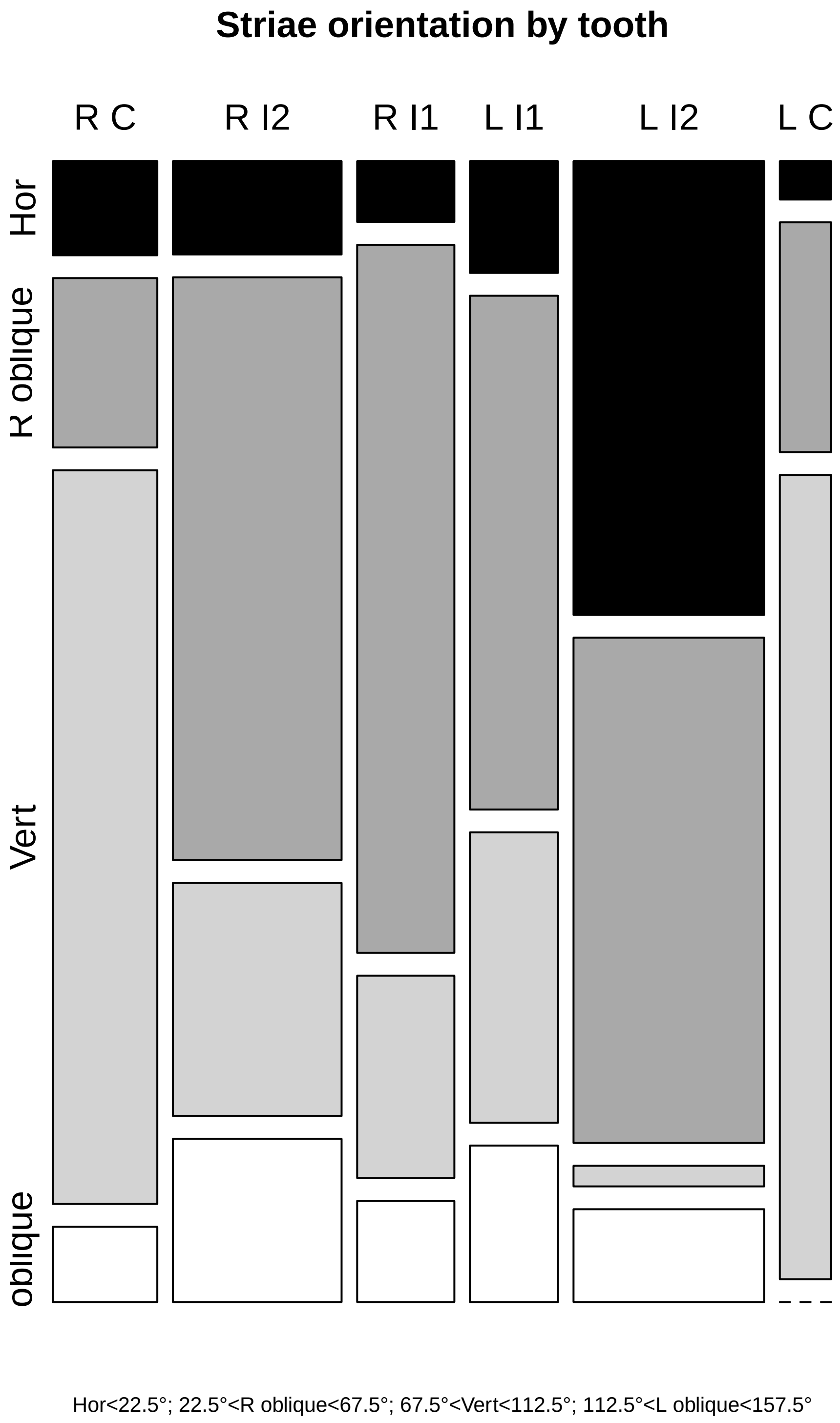
Striae orientation by tooth
R C
R I2
R I1
L I1
L I2
L C
Hor
R oblique
Vert
oblique
Hor<22.5°; 22.5°<R oblique<67.5°; 67.5°<Vert<112.5°; 112.5°<L oblique<157.5°
