# Supplementary material for: Hand to Mouth in a Neandertal: Right-Handedness in Regourdou 1
Source: PLoS One. 2012 Aug 22;7(8):e43949. doi: 10.1371/journal.pone.0043949 (PMC3425541; doi:10.1371/journal.pone.0043949)
Supplement: Text S1 — Methods. (DOC) [file pone.0043949.s010.doc]

**Methods**

Bi- three-dimensional analyses of the Regourdou 1 clavicles, humeri, radii and ulnae are based on a synchrotron radiation microtomographic record (SR-µCT) performed at the beamline ID 17 set at the European Synchrotron Radiation Facility, Grenoble, France (1, experiments SC-1587 and SC-1749). Differently from the elements of the right side, which are virtually intact, all those from the left side are incomplete to different degrees. In particular, the left humerus and radius are represented by two non-articulating pieces (2). To allow sufficiently detailed three-dimensional reconstructions and to perform reliable comparative linear, surfacial, and volumetric measurements, the final volumes of the microtomographic record were reconstructed in 8-bit format with an isotropic voxel size of 350 μm. Elaborations were run by AMIRA v4.0 package (Mercury Computer Systems, Inc.). Segmentation of the volumes were done semi-automatically with manual corrections. Whenever possible, cross-sectional geometric properties have been assessed at 35%, 50% (midshaft) and 65% of the length of each bone (3); however, because of their incompleteness, sections on the humerus and the radius were taken at 44% and 45%, respectively, instead that at the midshaft. The selected parameters considered in this study are: cortical area (CA), which measures the amount of compact bone in the diaphysis; polar second moment of area (J), which reflects the torsional strength of the shaft under loading (4, 5).

Cortical bone volume (CV) has been distinctly assessed for the distal (dCV, lateral for the clavicle) and the proximal (pCV, medial for the clavicle) portions of the diaphysis on the clavicle (dCV: 25-45%; pCV: 60-80%), the humerus (dCV: 25-44%; pCV: 60-80%), the radius (dCV: 25-45%; pCV: 61-80%), and the ulna (dCV: 25-45%; pCV: 60-80%), and the degree of bilateral asymmetry then calculated for each portion. Recent studies of torsional bending strength and cross-sectional bone area in humeral and ulnar midshafts of living males, show that the dominant limb shows significantly greater values than the opposite side (6).

Bilateral asymmetry has been measured as [(max – min)/min] x 100 (4); for the humerus, additional technical details can be found in (1).

Tooth impressions were made with RepliSet T-3, a high viscosity polyvinyl siloxane made by Struers Inc. The replicas were made with Epofix, an epoxy product also made by Struers Inc. All analyses were done on the Epofix replicas. Scratches only appear on the labial surface and are of two types: dietary scratches, recognized by their small size, very narrow width and shallowness (7) and manipulative scratches which are more distinct and seen by the eye. Tooth surfaces were digitally photographed with a binocular microscope using a digital camera. At 20-80x magnification striations were traced manually and saved in a vector format, which was used for measuring quantity, angle and scratch length. To avoid interobserver error, all striation identifications were done by one person (I.F.). After tracing the scratches the image was calibrated with mesial-distal length used to derive the pixels per cm (pcm) scale and the image was converted to black and white to enhance contrast. Then, the original background image was eliminated, leaving only the lines. We utilized NIH freeware ImageJ: version 1.4g, and the morphological particle analysis routine, enabling the calculation of many morphological parameters (among them length and angle) for each particle (the scratches in this case).

This software demanded no line crossovers, so we made two images --- one with the main striations and another with only crossover lines. These were tabulated separately by the software, and then combined in the statistical analysis, which counted the number of lines and calculated the length and angle of each line relative to the occlusal plane. Length showed no correlation with angle for any tooth (SI), so we did not consider it further. Data for each tooth were uploaded to a database for statistical analysis. Because ImageJ picks up small, insignificant portions of lines, all marks less than 0.1mm were eliminated. Scratch breadth and depth were not measured.

We document scratches following the intervals proposed by (8). Scratch angles were divided into four categories: horizontal (0-22.5°, >157.5-180°); right oblique (>22.5-67.5°); vertical (>67.5- 112.5°); left oblique (>112.5-157.5°). We tested the differences scratches with the null hypothesis that all scratches had the same probability to be in any orientation using chi^2^ (p<.01).

For SEM analysis, the transparent araldite replicas were sputter coated with gold by means of an Emitech K550X system. The SEM work was performed on a Zeiss EVO 60 housed in Rome at the Istituto Superiore per la Conservazione ed il Restauro.

**References**

1. Volpato V, Couture C, Macchiarelli R, Vandermeersch B (2011) Endostructural characterisation of the Regourdou 1 Neanderthal proximal arm: bilateral asymmetry and handedness. In: Condemi S, Weniger G-C, editors. Continuity and discontinuity in the peopling of Europe: One hundred fifty years of Neanderthal study. Vertebrate Paleobiology and Paleoanthropology. New York: Springer, pp.175-178.

2. Vandermeersch B, Trinkaus E (1995) The postcranial remains of the Régourdou I Neandertal: the shoulder and arm remains. J Hum Evol 28: 439-476.

3. Ruff C (2002) Variation in human body size and shape. Ann Rev Anthropol 31: 211-232.

4. Trinkaus E, Churchill SE, Ruff CB (1994) Postcranial robusticity in *Homo.* II: Humeral bilateral asymmetry and bone plasticity. Amer J Phys Anthropol 93: 1-34.

5. Ruff CB (2008) Biomechanical analyses of archaeological human skeletal samples. In: Katzenberg MA, Saunders SR, editors. Biological anthropology of the human skeleton, 2nd ed. Hoboken: Wiley-Liss, pp. 183-206.

6. Shaw C. (2011) Is ‘hand preference’ coded in the hominin skeleton. An in-vivo study of bilateral morphological variation. J Hum Evol 61:n480-487.

7. Ungar PS, Spencer MA (1999) Incisor microwear, diet, and tooth use in three Amerindian populations. Amer J Phys Anthropol 109: 387-396.

8. Bermúdez de Castro JM, Bromage TG, Fernández-Jalvo Y (1988) Buccal striations on fossil human anterior teeth: evidence of handedness in the middle and early Upper Pleistocene. J Hum Evol 17: 403-412.
