# Supplementary material for: Hand to Mouth in a Neandertal: Right-Handedness in Regourdou 1
Source: PLoS One. 2012 Aug 22;7(8):e43949. doi: 10.1371/journal.pone.0043949 (PMC3425541; doi:10.1371/journal.pone.0043949)
Supplement: Table S1 — Degree of bilateral asymmetry (%) in Regourdou 1 for the cortical area (CA) and the polar second moment of area (J) measured at three cross-sectional levels (distal / lateral, around the midshaft, proximal / medial) of the diaphysis on the clavicle (35%, 50%, 65%), the humerus (35%, 44%, 65%), the radius (35%, 45%, 65%), and the ulna (35%, 50%, 65%). (DOC) [file pone.0043949.s011.doc]

Table S1

Degree of bilateral asymmetry (%) in Regourdou 1 for the cortical area (CA) and the polar second moment of area (J) measured at three cross-sectional levels (distal / lateral, around the midshaft, proximal / medial) of the diaphysis on the clavicle (35%, 50%, 65%), the humerus (35%, 44%, 65%), the radius (35%, 45%, 65%), and the ulna (35%, 50%, 65%).

|  | distal /  lateral | midshaft | proximal /  medial | average |
| --- | --- | --- | --- | --- |
| **CA** |  |  |  |  |
| clavicle | 17.4 | 24.3 | 11.7 | 17.8 |
| humerus | 9.7 | 11.0 | 14.5 | 11.7 |
| radius | 8.6 | 6.2 | 10.4 | 8.4 |
| ulna | 19.8 | 15.9 | 13.0 | 16.2 |
|  |  |  |  |  |
| **J** |  |  |  |  |
| clavicle | 31.3 | 64.1 | 25.6 | 40.3 |
| humerus | 14.6 | 16.1 | 32.3 | 21.0 |
| radius | 17.5 | 17.8 | 33.8 | 23.0 |
| ulna | 45.4 | 38.0 | 32.2 | 38.5 |
