# Supplementary material for: Hand to Mouth in a Neandertal: Right-Handedness in Regourdou 1
Source: PLoS One. 2012 Aug 22;7(8):e43949. doi: 10.1371/journal.pone.0043949 (PMC3425541; doi:10.1371/journal.pone.0043949)
Supplement: Table S2 — Degree of bilateral asymmetry (%) in Regourdou 1 for the cortical bone volume (CV) distinctly assessed for the distal (dCV, lateral for the clavicle) and the proximal (pCV, medial for the clavicle) portions of the diaphysis on the clavicle (dCV: 25–45%; pCV: 60–80%), the humerus (dCV: 24–44%; pCV: 60–80%), the radius (dCV: 25–45%; pCV: 61–80%), and the ulna (dCV: 25–45%; pCV: 60–80%). (DOCX) [file pone.0043949.s012.docx]

Table S2

Degree of bilateral asymmetry (%) in Regourdou 1 for the cortical bone volume (CV) distinctly assessed for the distal (dCV, lateral for the clavicle) and the proximal (pCV, medial for the clavicle) portions of the diaphysis on the clavicle (dCV: 25-45%; pCV: 60-80%), the humerus (dCV: 25-44%; pCV: 60-80%), the radius (dCV: 25-45%; pCV: 61-80%), and the ulna (dCV: 25-45%; pCV: 60-80%).

|  | **dCV** | **pCV** |
| --- | --- | --- |
|  |  |  |
| clavicle | 23.1 | 18.8 |
| humerus | 8.3 | 15.6 |
| radius | 14.5 | 8.1 |
| ulna | 20.1 | 11.3 |
