# Supplementary material for: Hand to Mouth in a Neandertal: Right-Handedness in Regourdou 1
Source: PLoS One. 2012 Aug 22;7(8):e43949. doi: 10.1371/journal.pone.0043949 (PMC3425541; doi:10.1371/journal.pone.0043949)
Supplement: Table S3 — Summary straie statistics for all Regourdou 1 teeth (intervals according to [69] ). (DOCX) [file pone.0043949.s013.docx]

Table S3

Regourdou 1. Summary statistics for all teeth (intervals according to ref. 1)

|  | Horizontal  0° - 22.5°  >157.5° - 180° | Right oblique  >22.5°- 67.5° | Vertical  >67.5°- 112.5° | Left oblique  >112.5° - 157.5° | Total |
| --- | --- | --- | --- | --- | --- |
| Right canine | 5 | 9 | 39 | 4 | 57 |
| Right lateral incisor | 8 | 50 | 20 | 14 | 92 |
| Right central incisor | 3 | 35 | 10 | 5 | 53 |
| Left central incisor | 5 | 23 | 13 | 7 | 48 |
| Left lateral incisor | 44 | 49 | 2 | 9 | 104 |
| Left canine | 1 | 6 | 21 | 0 | 28 |
| Total | 66 | 172 | 105 | 39 | 382 |

Pearson's Chi-squared test with simulated p-value (based on 5000 replicates)

X-squared = 165.8339, df = NA, p-value = 2e-04

Chi-squared test for equal probability with simulated p-value (based on 5000 replicates)

data:

| Right canine | 5 | 9 | 39 | 4 |
| --- | --- | --- | --- | --- |

X-squared = 58.2982, df = NA, p-value = 2e-04

Chi-squared test for equal probability with simulated p-value (based on 5000 replicates)

data:

| Right lateral incisor | 8 | 50 | 20 | 14 |
| --- | --- | --- | --- | --- |

X-squared = 45.3913, df = NA, p-value = 2e-04

Chi-squared test for equal probability with simulated p-value (based on 5000 replicates)

data:

| Right central incisor | 3 | 35 | 10 | 5 |
| --- | --- | --- | --- | --- |

X-squared = 49.566, df = NA, p-value = 2e-04

Chi-squared test for equal probability with simulated p-value (based on 5000 replicates)

data:

| Left central incisor | 5 | 23 | 13 | 7 |
| --- | --- | --- | --- | --- |

X-squared = 16.3333, df = NA, p-value = 0.0012

Chi-squared test for equal probability with simulated p-value (based on 5000 replicates)

data:

| Left lateral incisor | 44 | 49 | 2 | 9 |
| --- | --- | --- | --- | --- |

X-squared = 66.0769, df = NA, p-value = 2e-04

Chi-squared test for equal probability with simulated p-value (based on 5000 replicates)

data:

| Left canine | 1 | 6 | 21 | 0 |
| --- | --- | --- | --- | --- |

X-squared = 40.2857, df = NA, p-value = 2e-04

1. Bermúdez de Castro JM, Bromage TG, Fernández-Jalvo Y (1988) Buccal striations on fossil human anterior teeth: evidence of handedness in the middle and early Upper Pleistocene. J Hum Evol 17: 403-412.
